# Comparison of the Prognostic Usefulness of the European Society of Cardiology and American Heart Association/American College of Cardiology Foundation Risk Stratification Systems for Patients With Hypertrophic Cardiomyopathy

**DOI:** 10.1016/j.amjcard.2017.10.027

**Published:** 2018-02-01

**Authors:** Kevin M.W. Leong, Ji-Jian Chow, Fu Siong Ng, Emanuela Falaschetti, Norman Qureshi, Michael Koa-Wing, Nicholas W.F. Linton, Zachary I. Whinnett, David C. Lefroy, D. Wyn Davies, Phang Boon Lim, Nicholas S. Peters, Prapa Kanagaratnam, Amanda M. Varnava

**Affiliations:** a National Heart & Lung Institute, Imperial College, London, United Kingdom; b Imperial College Healthcare NHS Trust, London, United Kingdom

## Abstract

Implantable cardiodefibrillators (ICDs) have proven benefit in preventing sudden cardiac death (SCD) in hypertrophic cardiomyopathy (HC), making risk stratification essential. Data on the predictive accuracy on the European Society of Cardiology (ESC) risk scoring system have been conflicting. We independently evaluated the ESC risk scoring system in our cohort of patients with HC from a large tertiary center and compared this with previous guidance by the American College of Cardiology Foundation and Heart Association (ACCF/AHA). Risk factor profiles, 5-year SCD risk estimates, and ICD recommendations, as defined by the ACCF/AHA and ESC guidelines, were retrospectively ascertained for 288 HC patients with and without SCD or equivalent events at our center. In the SCD group (n = 14), a significantly higher proportion of patients would not have met the criteria for an ICD implant using the ESC scoring algorithm compared with ACCF/AHA guidance (43% vs 7%, p = 0.029). In those without SCD events (n = 274), a larger proportion of individuals not requiring an ICD was identified using the ESC risk score model compared with the ACCF/AHA model (82% vs 57%; p < 0.0001). Based on risk stratification criteria alone, 5 more individuals with a previously aborted SCD event would not have received an ICD with the ESC risk model compared with the ACCF/AHA risk model. In conclusion, we found that the current ESC scoring system potentially leaves more high-risk patients unprotected from sudden death in our cohort of patients.

Hypertrophic cardiomyopathy (HC) affects 1 of 500 adults in the general population and carries a risk of sudden cardiac death (SCD).^1^ Although the implantable cardiodefibrillator (ICD) has proven efficacy in treating lethal ventricular arrhythmias and preventing SCD,^2^, ^3^ they are not without complication.^4^ Consequently, management of HC requires accurate risk assessment in all patients and ICDs recommended in those deemed to be of sufficient risk. The 2003 American College of Cardiology/European Society of Cardiology (ESC) and the 2011 American Heart Association (AHA)/American College of Cardiology Foundation (ACCF) guidance identify high-risk patients based on 5 risk factors.^5^, ^6^ Despite the positive correlation of risk to the number of factors, it was suggested that the discriminating ability, between low- and high-risk patients, of the 2003/2011 guidance could be improved upon.^7^, ^8^ In 2014, the ESC endorsed a novel risk scoring model that provided an individualized estimate of 5-year SCD risk.^7^, ^9^ The predictive accuracy of this tool has been assessed by 2 other studies but results have been conflicting.^10^, ^11^, ^12^ These studies evaluating this model had also excluded patients with previous SCD events, and it is unclear if the model would have recommended an ICD implant had they been seen or screened earlier.^10^, ^11^, ^12^ As these individuals are also part of the high-risk cohort the model was designed to identify, we investigate if the model would have recommended ICD implants in these individuals before their presenting SCD event. We also explore the impact different scoring systems have on ICD recommendations in this retrospective study of our cohort of patients.

## Methods

The study population consisted of patients with HC aged above 16 years currently under follow-up at Imperial College Healthcare NHS Trust, a large tertiary care center in London. The diagnosis of HC was based on echocardiographic findings of ≥15 mm of unexplained left ventricular hypertrophy (LVH).^13^ Patients with LVH associated with Fabry disease, amyloidosis, mitochondrial disease, or congenital heart defects were excluded. As the aim of the study was to retrospectively assess the predictive accuracy of recommendations using different guidelines, we also included patients with HC whose initial presentation was an SCD event and survived. Predictive risk scores and ICD implant recommendations based on the ESC 2014 and AHA/ACCF 2011 and 2003 guidelines were determined by assessing the case records in all these patients. A total of 312 patients were identified and 24 were excluded from the study owing to missing data variables that would not permit calculation of the ESC 5-year risk score. Approval of the study protocol and design was obtained by the local institutional review board.

The risk factors and variables considered were those described in previous guidance.^5^, ^6^, ^7^, ^9^ These included (1) age at evaluation, (2) family history of SCD in ≥1 first-degree relatives aged <40 years or in a first-degree relative with confirmed HC at any age, (3) left ventricular wall thickness, (4) history of unexplained syncope, (5) documented nonsustained ventricular tachycardia (NSVT) defined as ≥3 beats at a rate of ≥120 beats/min, (6) maximal left ventricular outflow tract (LVOT) gradient, and (7) left atrial diameter. LVH ≥ 30 mm, LVOT gradient ≥ 30 mm Hg, abnormal blood pressure response during exercise, presence of coexisting atrial fibrillation (AF), and presence of fibrosis on magnetic resonance imaging were also obtained to allow calculation of the number of conventional and minor risk factors considered in the 2003 and 2011 guidelines.

Recommendation for ICD implant was separated into 3 categories—‘recommended,’ ‘considered,’ and ‘not recommended.’ The ‘recommended’ category included individuals who had a 5-year SCD risk score >6% based on the 2014 guidance, or ≥1 conventional risk factor based on the 2011 guidance, or ≥2 risk factors based on the 2003 guidance. The ‘considered’ category included individuals with a 5-year risk score between 4% and 6%, or presence of NSVT or abnormal blood pressure response during exercise alone with the presence of other minor risk factors outlined in 2011 guidance, or the presence of ≥1 risk factor based on the 2003 guidance. In those where an ICD would ‘not be recommended’, individuals had a 5-year risk score <4% under the 2014 model or an absence of any of the 5 risk factors under the 2011/2003 guidance.

SCD risk score calculation was performed at baseline evaluation in line with previous studies.^9^, ^10^, ^11^ For patients whose first presentation was an SCD event, risk factor assessment and score calculation for age were based on point of event and on clinical investigations obtained up to a year after event for the other variables. A history of syncope in these patients was deemed as a factor if the patients had it at any point before their presenting SCD event. End points for all other patients was an SCD or equivalent event, which included (1) resuscitation from cardiac arrest or (2) having received an appropriate ICD discharge in response to ventricular fibrillation or fast ventricular tachycardia (>200 beats/min).^9^, ^10^, ^11^ Follow-up was from first evaluation to an SCD end point or to the censure date set as October 1, 2016. For those lost to follow-up, this was until their last known contact date.

Appropriate and inappropriate ICD therapies were also retrieved from patient case records. In all patients with reported ICD therapy, intracardiac electrograms from the device were evaluated to exclude ICD discharges in response to an accelerated ventricular rhythm caused by antitachycardic pacing (ATP) delivered by the ICD.

Categorical variables are presented as percentages and continuous data as mean ± standard deviation. Differences between groups were performed using Student *t* test for continuous variables and chi-square test for categorical variables. Receiver operating characteristic (ROC) analysis was used to evaluate the diagnostic performance, that is, the ability to discriminate between SCD and non-SCD cases of the different risk stratification methods. ROC curves, a graph of the sensitivity versus 1 − specificity of the diagnostic test, were presented, and the areas under the ROC curve (or C-index) were calculated. The equality of the areas was tested using the DeLong method. A p value of <0.05 was considered statistically significant. Stata statistical software package (Release 14; StataCorp LLC, Texas) was used for statistical analysis.

## Results

The final study population comprised 288 patients (mean age 52 ± 16 years; 66% male) with clinical characteristics summarized in Table 1. Increased LVOT gradient (≥30 mm Hg) was present in 73 patients (25% of the cohort). Exercise treadmill test and magnetic resonance imaging data were available in 184 and 195 patients, respectively. A total of 75 patients (26%) received an ICD for primary (n = 66) and secondary (n = 9) prevention. Eleven patients from the primary prevention group received ATP and/or shock therapy from their ICD during follow-up. The mean follow-up period was 5.6 ± 3.8 years.

**Table 1:** Group characteristics of hypertrophic cardiomyopathy cohort

	All (n = 288)	Patients with previous aborted sudden cardiac death or equivalent events (n = 14)	Patients without previous aborted sudden cardiac death or equivalent events (n = 274)	p-value*
Male	191 (66%)	10 (71%)	181 (66%)	0.68
Age (years)	52 ± 16	41 ± 18	53 ± 15	*<0.01*
Syncope	34 (12%)	5 (36%)	29 (11%)	*<0.01*
Family history of sudden cardiac death	40 (14%)	9 (64%)	31 (11%)	*<0.001*
Maximal left ventricular wall thickness ≥30 mm	16 (6%)	0	16 (6%)	0.35
Left ventricular hypertrophy thickness (mm)	20 ± 5	22 ± 4	20 ± 5	0.14
Non-sustained ventricular tachycardia	66 (23%)	9 (64%)	5 (2%)	*<0.001*
Abnormal blood pressure response to exercise	32/184 (17%)	1/7 (14%)	31/177 (18%)	0.83
Maximal left ventricular outflow tract gradient (mmHg)	28 ± 41	17 ± 26	28 ± 42	0.33
Left atrial diameter (mm)	41 ± 7	41 ± 8	41 ± 7	1.00
Late gadolinium enhancement on cardiac magnetic resonance imaging	127/195 (65%)	4/5 (80%)	123/190 (65%)	0.48
Atrial fibrillation	57 (20%)	6 (43%)	47 (17%)	*0.02*
0 risk factors	158 (55%)	1 (7%)	157 (57%)	*<0.001*
1 risk factors	81 (28%)	5 (36%)	76 (28%)	0.52
≥2 risk factors	49 (17%)	8 (57%)	41 (15%)	*<0.0001*

Values are presented in absolute numbers (percentages) and mean ± standard deviation.

* Comparison between patients with and without previous aborted sudden cardiac death or equivalent events.

Fourteen patients experienced an SCD or equivalent event. This comprised 9 individuals who presented to our service after successful out-of-hospital cardiac resuscitation and 5 who received appropriate ICD shock therapy over the follow-up. The SCD cohort had a higher proportion of patients with previous syncope, NSVT, family history of SCD, and AF than those without (Table 1). Six other patients received appropriate ATP pacing therapy for sustained ventricular arrhythmias.

**Figure 1:**
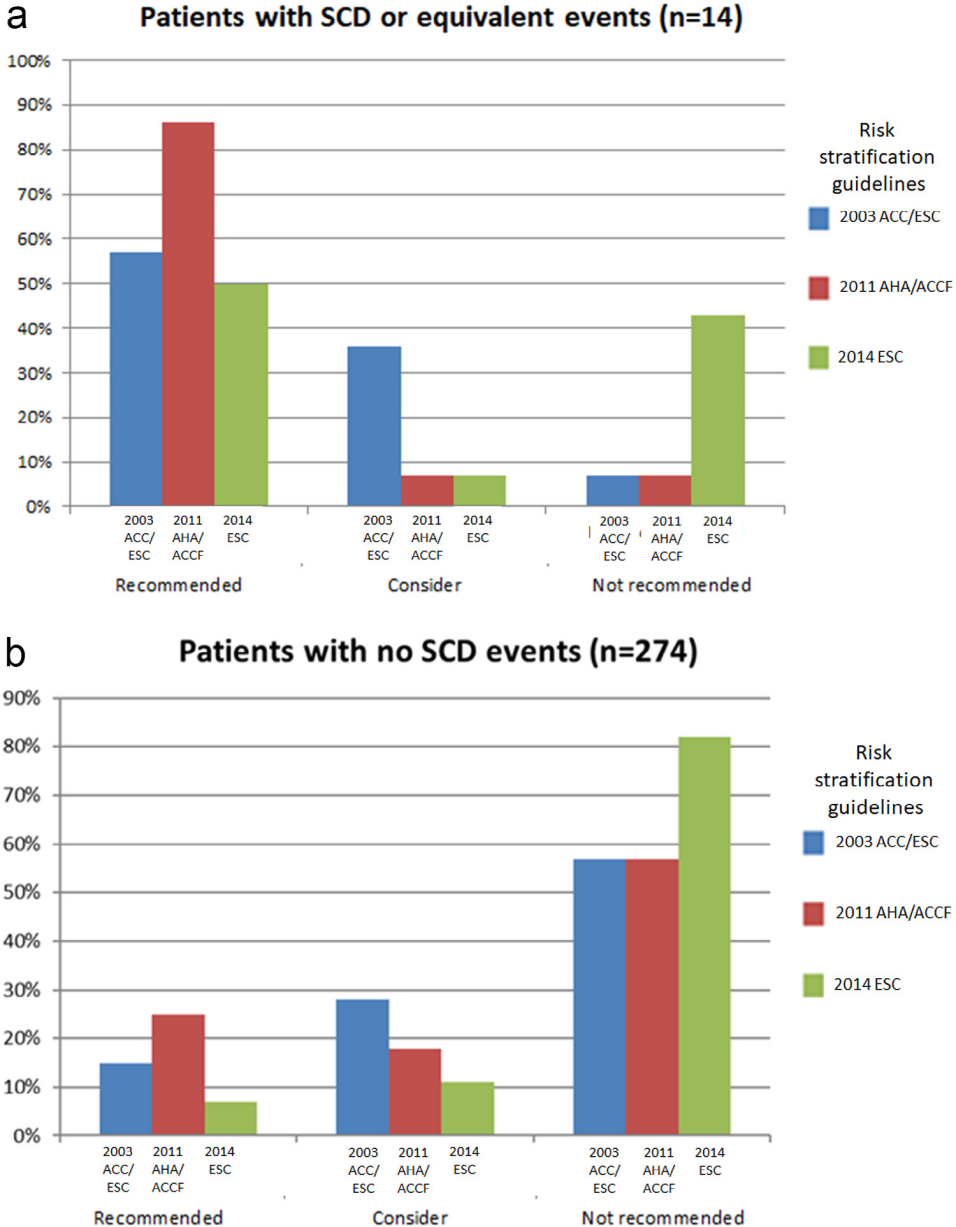
Proportion of patients grouped according to ICD recommendations by the 2014 *(green)*, 2011 *(red)*, and 2003 *(blue)* models in the sudden cardiac death group *(A)* and non-sudden cardiac death group *(B)*. ACC = American College of Cardiology; ACCF = American College of Cardiology Foundation; AHA = American Heart Association; ESC = European Society of Cardiology. (Color version available online.)

The comparative differences between the 2014 and the 2011/2003 guidance in ICD implant recommendation within the SCD group are summarized in Table 2. There was a larger proportion of patients in whom an ICD would not have been recommended using the 2014 scoring criteria (<4%/5 years) compared with AHA 2011 and 2003 guidance (43% vs 7%, p = 0.029 and 43% vs 7%, p = 0.029, respectively) (Figure 1). In a subgroup analysis of the SCD group, after excluding those who received an ICD for secondary prevention, a greater proportion of individuals (3 of 5 [60%]) would similarly not have met the recommendation for an ICD based on the risk score model compared with the 2011/2003 model (1 of 5 [20%]). If we included 6 additional patients who received appropriate ATP therapy for sustained VT into the SCD group, the 2014 risk model would not have identified a larger proportion for an ICD compared with the 2011/2003 guidance (2014 vs 2011: 45% vs 5%, p = 0.0035; 2014 vs 2003: 45% vs 5%, p = 0.0035) (Table 3).

**Table 2:** ICD recommendations using different risk scoring systems in patients with previous sudden cardiac death or equivalent events

Patients with sudden cardiac death or equivalent events (n = 14)					
ICD Guidance	ACC/ESC 2003	ACCF/AHA 2011	ESC 2014	2014 vs 2003	2014 vs 2011
Recommended	8 (57%)	12 (86%)	7 (50%)	−7% (−44%, 30%)	−37% (−68%, −4%)
Consider	5 (36%)	1 (7%)	1 (7%)	−29% (−57%, −0.1%)	0%
Not recommended	1 (7%)	1 (7%)	6 (43%)	36% (6%, 65%)	36% (6%, 65%)

Values are presented in absolute numbers (percentages). Comparisons are presented as mean differences (95% confidence interval).

**Table 3:** ICD recommendations using different risk scoring systems in patients with previous sudden cardiac death events and appropriate ICD therapy

Patients with sudden cardiac death events and all appropriate ICD therapy (n = 20)					
ICD Guidance	ACC/ESC 2003	ACCF/AHA 2011	ESC 2014	2014 vs 2003	2014 vs 2011
Recommended	12 (60%)	16 (80%)	8 (40%)	−20% (−50%, 10%)	−40% (19%, 61%)
Consider	7 (35%)	3 (15%)	3 (15%)	−20% (−46%, 6%)	0%
Not recommended	1 (5%)	1 (5%)	9 (45%)	40% (16%, 64%)	40% (16%, 64%)

Values are presented in absolute numbers (percentages). Comparisons are presented as mean differences (95% confidence interval).

A total of 274 patients with HC did not experience an SCD or receive shock therapy for an SCD equivalent event. Table 4 summarizes the proportions according to ICD implant recommendations using different guidelines. A larger proportion of individuals not requiring an ICD was identified using the 2014 risk score model compared with the 2011/2003 models (82% vs 57%; p < 0.0001) (Figure 1). It was also observed that the 2003 and 2011 guidance would have recommended a greater majority of these patients for an ICD (n = 117, 43%) compared with the ESC risk score which identified a much lower proportion (n = 50, 18%).

**Table 4:** ICD recommendations using different risk scoring systems in patients without previous sudden cardiac death or equivalent events

Patients without sudden cardiac death or equivalent events (n = 274)					
ICD Guidance	ACC/ESC 2003	ACCF/AHA 2011	ESC 2014	2014 vs 2003	2014 vs 2011
Recommended	41 (15%)	68 (25%)	21 (7%)	−7% (−13%, −2%)	−17% (−23%, −11%)
Consider	76 (28%)	49 (18%)	29 (11%)	−17% (−24%, −11%)	−7% (−13%, −1%)
Not recommended	157 (57%)	157 (57%)	224 (82%)	24% (17%, 32%)	24% (17%, 32%)

Values are presented in absolute numbers (percentages). Comparisons are presented as mean differences (95% confidence interval).

During the follow-up period, 6 individuals experienced inappropriate therapies and were all from the non-SCD group. Two of the inappropriate therapies related to T wave oversensing with 1 requiring an explant of his subcutaneous ICD for repeat shocks despite reprogramming, and 4 were related to supraventricular arrhythmias. Under the 2003/2011 guidance, 5 of these individuals would have required an ICD based on recommendations. In comparison, only 1 would have met a clear requirement for an ICD (>6%) and 4 where an ICD could be considered (4% to 6%) under the 2014 guidelines.

**Figure 2:**
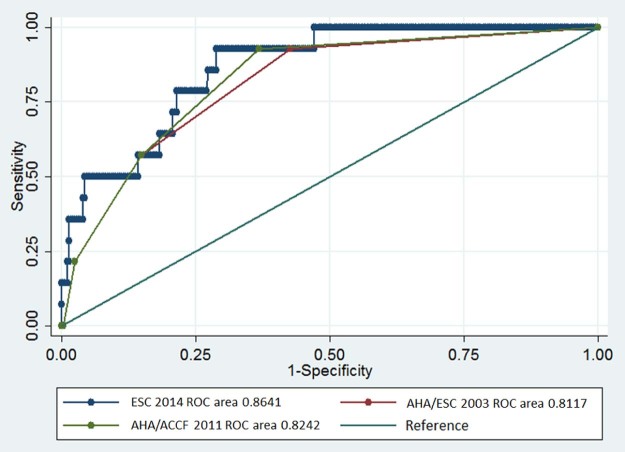
Receiver operating characteristic curves for the risk prediction models of the 2014 ESC, 2011 AHA/ACCF, and 2003 ACC/ESC guidelines, and the reference line (area under curve = 0.5). ACC = American College of Cardiology; ACCF = American College of Cardiology Foundation; AHA = American Heart Association; ESC = European Society of Cardiology. (Color version available online.)

The SCD group had a mean calculated 5-year risk of 7.5 ± 5.6% and a median of 2 risk factors. In patients without SCD events, the mean 5-year risk score was 2.7 ± 2.1% and median number of risk factors was 0. The area under the ROC curve or C-index for the 2014 risk score model was 0.86 (95% confidence interval [CI] 0.78 to 0.94; p < 0.0001), which was higher than the C-index calculated for the 2003 (0.82 [95% CI 0.72 to 0.93]; p < 0.0001) or 2011 guidelines (0.83 [95% CI 0.73–0.93]; p < 0.0001). Their respective ROC curves were plotted and are shown in Figure 2. Using the thresholds outlined previously,^5^, ^6^, ^7^ sensitivity and specificity were 57% and 82%, respectively, with the 2014 model, 93% and 64% with the 2011 guidance, and 93% and 57% with the 2003 guidance.

We also determined the potential impact a change in risk stratification strategy would have on ICD recommendations in our cohort. In those with SCD events, 5 of this group (36%) would have had a downgrade in ICD recommendation when moving from 2011 to 2014 guidance. Majority (64%) would not have had a change in recommendation. Within the non-SCD group, 82 (30%) would have had a downgrade, whereas 3 (1%) would have had an upgrade in recommendations. The majority in this group (69%) would not have seen a change in their recommendations. Table 5 summarizes the changes from the 2003/2011 guidance to the new 2014 model.

**Table 5:** Reclassification of ICD recommendations from 2003/2011 to 2014 risk model

	ICD recommendation downgraded with 2014 model	ICD recommendation not changed	ICD recommendation upgraded with 2014 model	Total
**2003 vs 2014 guidelines**				
Patients with previous sudden death events	5 (36%)	8 (57%)	1 (7%)	14
Patients without previous sudden death events	78 (29%)	190 (69%)	6 (2%)	274
**2011 vs 2014 guidelines**				
Patients with previous sudden death events	5 (36%)	9 (64%)	0	14
Patients without previous sudden death events	82 (30%)	189 (69%)	3 (1%)	274

## Discussion

Compared with the 2011/2003 model, our study found that the 2014 risk score algorithm has the potential of leaving more patients vulnerable to SCD without an ICD. Further, 43% (n = 6) of our SCD patients would not have received an ICD with the 2014 model, which lowered to 7% (n = 1) with the 2011/2003 model. In other words, 5 more individuals who experienced an aborted SCD event would not have received an ICD recommendation (Table 5) when moving from the 2011/2003 to the 2014 risk model. Similarly, Maron et al reported a much greater proportion of SCD patients who would not have achieved a recommendation for an ICD implant with the risk score algorithm compared with the 2011 model (59% vs 12%). Even though Vriesendorp et al demonstrated that a lower proportion of the SCD group would not have been recommended for an ICD implant based on the 2014 risk score model compared with the 2011 model (30% vs 50%), this is not an insignificant proportion and their analysis was limited to half of their total SCD cohort (20 of 41).^11^ Although it is important to minimize the need for ‘unnecessary’ ICD implants as this has been known to lead to morbidity and mortality, the primary goal of any risk stratification system is to identify the high risk and prevent SCD.

The risk factors and criteria for ICD recommendation in the AHA/ACCF 2011 guidance are also supported by several survival and large comparative cohort studies.^13^, ^14^, ^15^, ^16^, ^17^, ^18^, ^19^, ^20^ Although it has been suggested that a larger proportion of individuals without a future SCD event would have received an ICD based on this guidance,^8^, ^11^ a long-term follow-up study involving 1,000 patients demonstrates the effectiveness of such a strategy in reducing the SCD burden in HC.^21^ Contemporary treatment and interventions based on using ≥1 conventional risk markers have reduced mortality rates across all age groups to <1%/year compared with the mortality rate that ranged from 1.5% to 6%/year before the introduction of the 2011 risk model.^22^, ^23^ Given the relatively recent formulation of the ESC risk scoring algorithm, equivalent long-term outcome data with this model have yet to be published.

To compare the prognostic utility of different risk stratification systems, one may use the C-index, or concordance index, score that provides a statistical measure of how well a tool correctly predicts the outcome of an individual sampled at random. The 2014 ESC risk algorithm has been reported to possess a higher C-index score compared with the 2011/2003 guidance previously,^8^, ^9^, ^11^ but this may not always indicate a higher sensitivity *and* specificity. In our group of patients, we found that a greater proportion of the SCD group would not have had an ICD implant recommended using the 2014 ESC risk score calculator (low sensitivity). At the same time, it correctly identified a much larger proportion of individuals who would not have needed an ICD (higher specificity), which accounted for the larger C-index score it achieved over the 2011/2003 model.

The reported sensitivity and specificity of the 2014 risk scoring algorithm in the literature have also been different from our findings and variable.^9^, ^10^, ^11^, ^12^ In the original cohort, 3,675 patients analyzed by the HC Outcome Investigators, the sensitivity and specificity of the 2014 scoring algorithm were originally reported at 71% and 70%, respectively,^9^ and found to be similar in a separate evaluation of 706 patients (70% and 67%).^11^ Differences in SCD cohort characteristics, such as a greater proportion of patients with NSVT and family history of SCD (Table 6), and the inclusion of previous SCD cases may account for the differences in sensitivity and specificity seen in our results (57% and 82%, respectively). However, Maron et al conducted a study in 1,629 HC individuals and reported a much lower sensitivity of 9%, but higher specificity of 96%,^10^ despite similarities in cohort characteristics to the previous 2 studies^9^, ^11^ (Table 6). In another study of 135 patients with HC, lower sensitivity and specificity (66% and 42%) were reported.^12^ The wide variability seen in sensitivity and specificity in these studies belies the notion that current risk stratification techniques are reliable and underscores the need to further evaluate different HC cohorts with such criteria.

**Table 6:** Characteristics patients with previous aborted sudden death or equivalent events in other studies

	Our cohort (n = 14)	Vriesendorp et al (n = 42)	O'Mahony et al (n = 198)	Maron et al (n = 81)
Male	10 (71%)	32 (71%)	142/198 (72%)	50 (62%)
Average age (years)	41 ± 18	44 ± 17	43 ± 15	39 ± 15
Syncope	5 (36%)	7 (17%)	52 (26%)	21 (26%)
Family history of sudden cardiac death	9 (64%)	14 (33%)	73 (37%)	26 (32%)
Left ventricular wall thickness ≥30 mm	0	8 (19%)	n/a	18 (22%)
Maximal left ventricular wall thickness (mm)	22 ± 4	23 ± 5	22 ± 6	n/a
Non-sustained ventricular tachycardia	9 (64%)	16 (38%)	62 (31%)	20 (25%)
Abnormal blood pressure response to exercise	1/7 (14%)	5 (12%)	n/a	14 (17%)
Maximal left ventricular outflow tract gradient (mmHg)	17 ± 26	48 ± 43	18 (6-58)*	n/a
Left atrial diameter (mm)	41 ± 8	49 ± 9	46 ± 9	n/a

Data provided in absolute numbers (percentages) and mean ± standard deviation.

* Median (range) data only available.

The threshold for consideration of an ICD was set at a 5-year SCD risk of ≥4% and was based on being able to detect 71% of patients who would reach an SCD end point.^9^ In other words, we would expect 29% of these patients to fall below this threshold, or be missed, based on the original modeling study. One must therefore be cautious about reassuring patients who fall below this threshold. The results from our study and that of Maron et al also show a higher-than-expected proportion of patients who experienced an SCD event would have been left vulnerable.^10^

Ultimately, the ESC scoring system appears to have a higher C-index score because of its greater specificity in our cohort of patients. However, this may be at the cost of reduced sensitivity and thus missed opportunity to offer appropriate ICD therapy. In the end, either risk scoring system will provide an estimate of risk for the individual, which provides a useful anchor to base a discussion on the perceived risk and benefits of an ICD. However, an appreciation of the limitations of any risk scoring system is essential so that clinicians understand the potential implications of the quantitative measure of risk that is assigned.

There are considerations that have to be given to our dataset. In the retrospective calculation of baseline risk for those who presented with SCD events, scoring variables were obtained from workup performed at the point of SCD event. We also took into account data obtained up to a year after event (e.g. Holter for NSVT) in assessment of the SCD group. This was performed to minimize inadvertent underestimation of risk and does bias our results toward a higher estimation of SCD risk being calculated in this group. The numbers in our study are small, with data being prone to type I error. However, these data provide individual patient validation and real-world implications of using different risk scoring systems for each patient.

## Conclusion

We provide an independent and comparative evaluation of the different risk scoring systems. In our cohort, we found the 2014 scoring system has the ability to correctly identify low-risk individuals but potentially leaves more patients vulnerable to SCD without an ICD in comparison to previous risk stratification strategies.

## Disclosures

The authors have no conflicts of interest to disclose.
